# No Free Lunch With Herbal Preparations: Lessons From a Case of Parkinsonism and Depression Due to Herbal Medicine Containing Reserpine

**DOI:** 10.3389/fneur.2019.00634

**Published:** 2019-06-18

**Authors:** Michel Rijntjes, Philipp T. Meyer

**Affiliations:** ^1^Department of Neurology and Neurophysiology, Medical Center, Faculty of Medicine, University of Freiburg, Freiburg, Germany; ^2^Department of Nuclear Medicine, Medical Center, Faculty of Medicine, University of Freiburg, Freiburg, Germany

**Keywords:** Parkinson, depression, reserpine, VMAT, ayurvedic, Rauwolfia, complementary therapies

## Abstract

The increasing use of herbal medicines calls for a heightened awareness of their potential side-effects. This especially pertains to western countries, where patients tend to use herbal medicine as self-medication, often alongside regular prescriptions, and physicians have less experience with their application. Here we report a case in which Parkinsonism, depression, and an atypical finding detected by dopamine transporter single-photon emission computed tomography were all belatedly recognized as side-effects of herbal medicine. This only occurred because one of its active ingredients, reserpine, has been extensively studied. For most other herbal medicines, however, knowledge about side-effects remains scarce or unavailable. Therefore, we suggest that physicians, when taking a medication history, should actively ask for the use of any herbal preparations.

## Introduction

Of all forms of traditional and complementary medicine (T&CM), herbal medicines are the most popular. The prevalence of herbal medicine use in Africa is about 80% (1), in China 30–50% (2), in the USA 35% (3), and in Europe around 20%, although the latter varies considerably at the local level (4).

In less-developed countries, herbal medicines are used either as first-line therapy for patients who do not have access to expensive pharmaceuticals, or for traditional reasons, some of which date back to thousands of years, such as Ayurvedic medicine. Many herbs contain medicinal ingredients (5) and numerous pharmaceuticals (e.g., acetylsalicylic acid, digitalis, quinine, curare, morphine) are derived from plants first used in a traditional setting.

In western countries, herbal medicines are often taken for chronic diseases such as obesity, hypertension, cardiovascular disease, type II diabetes, cancer, arthritis, and depression, conditions for which conventional therapy does not guarantee complete remission (3, 6). Largely, these are “diseases of affluence,” associated with a long life-span, sedentary lifestyle, and high-calorie intake of processed food. Since pharmaceuticals are also product of an industrialized society, people may have developed a romantic view that ancient herbal medicines are a more “natural” way of treating diseases, and may further assume that there are no side effects because they are available without prescription. Moreover, suffering from a disease in today's competitive culture can lead to a feeling of failure, and patients may want to demonstrate their independence by managing their health problems on their own (6). Therefore, the vast majority of patients in western countries take herbal medicines as self-medication, often in addition to prescriptions medicines, without always providing this information to their physician.

We describe the case of a patient suffering from progressive Parkinsonism and depression for 3 years. Neither the patient, nor the general practitioner or the neurologist (M.R.) initially deemed the herbal tablets he took for hypertension as relevant. However, after the significance of the herbal medication was realized, it was discontinued and the patient recovered completely.

### Case Presentation

A 56-year old German man presented with bilateral, left-dominant rest, and postural hand tremor that first manifested ~2.5 years earlier and increased progressively over time. Physical examination revealed general bradykinesia, rigor of the left arm with dysdiadochokinesia, and diminished amplitude in tapping tasks with the left hand and foot. There was slight hypomimia but no slurred speech, hypophonia, or dyskinesia. The trunk was bent to the left when standing (Pisa-syndrome), left arm swing was diminished while walking with normal step length, there was no postural instability or history of falls. In addition, the patient developed less of an interest in social interactions and reported that he had become less decisive in his job as a lawyer, something which was also noticed by his colleague. His mood was slightly depressed without morning lows, his appetite had decreased but was not accompanied by weight loss and he woke up 1–2 h earlier than usual, albeit without ruminating thoughts. The MDS-UPDRS-III (Movement Disorder Society Unified Disease Rating Scale part III) was 18 and the BDI (Beck Depression Inventory) was 15. He denied other non-motor symptoms associated with Parkinson's disease, such as hyposmia, obstipation, and REM-sleep behavior disorder. His alcohol consumption was moderate and he had never smoked or taken illegal drugs.

For a duration of around 3 years, the patient took herbal tablets for hypertension, but no prescription drugs. His blood pressure was within the normal range. Both the routine blood examination that included copper metabolism and an MRI of the brain, prompted by his general physician, were unremarkable. There was no family history of movement disorders.

**Figure 1:**
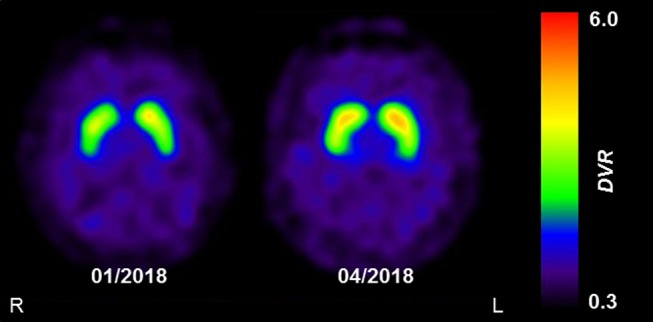
The effect of reserpine withdrawal on striatal DAT availability. Parametric images of striatal dopamine transporter (DAT) availability acquired using DAT-SPECT with [^123^I]FP-CIT. Images show the distribution volume ratio (DVR), which was used as a measure of DAT availability. The mild and homogenous decrease in DAT availability at initial presentation (01/2018) was completely resolved at follow-up (04/2018), 3 months after the withdrawal of reserpine. Images were acquired on a dual-headed SPECT system, 3 h after intravenous injection of 185 MBq [^123^I]FP-CIT.

The clinical presentation was deemed consistent with that of Parkinson's disease (PD). However, because non-motor symptoms other than a depressive mood were absent and ultrasound showed normal echogenicity of the substantia nigra [thus ruling out local PD-associated gliosis (7)], dopamine transporter (DAT) single-photon emission computed tomography (SPECT) with [^123^I]FP-CIT was performed to confirm PD. DAT SPECT showed a mild but remarkable bilateral homogenous reduction of DAT availability in the striatum (Figure 1; approximated striatal binding potential, BP: right 1.72, left, 1.89, lower normal cut-off: 2.1), without the anterior-posterior gradient with strong putaminal involvement that is typical for PD. This led to the suspicion of a systemic effect (e.g., pharmacological blockade).

On further questioning, the patient revealed that the Ayurvedic medication originally prescribed to him 3 years earlier for hypertension whilst on holidays in India was normalin®, one tablet twice a day. One year later, the patient visited the same Ayurvedic physician, who subsequently exchanged normalin® for serpina®, two tablets a day, and additionally prescribed tablets containing ashwagandha and brahmi. Six months preceding the first visit, the patient had increased the dose of serpina® to four tablets a day. Both normalin® and serpina® contain root extract from the plant Rauwolfia serpentina which is rich in reserpine, an inhibitor of the presynaptic uptake of neurotransmitters, such as dopamine and serotonin. Since the patient's symptoms appeared a few months after taking normalin® and progressed after switching to serpina®, we advised him to stop taking the herbal medication immediately.

After 1 month, there were no changes in the patient's symptoms, physical examination, MDS-UPDRS-III or BDI. He tried taking levodopa/benzerazide 100 mg/25 mg for 1 week and escitalopram 10 mg for 4 weeks, but since this had no effect on his bradykinesia or depressive mood, he discontinued this medication.

Two months after discontinuing the herbal medication, the patient's mood had improved substantially and his motivation for working and socializing had returned to the previous normal levels, the BDI was zero. His hypertension was controlled with 8 mg candesartan daily. All motor symptoms had vanished except for a persistent positional tremor in the left hand. After 3 months, this tremor had also disappeared and a follow-up DAT SPECT scan had normalized completely (Figure 1; striatal BP: right, 2.22; left, 2.34). Another 12 months later, none of the symptoms had reappeared and he felt himself to be completely healthy. The patient was ultimately diagnosed with transient Parkinsonism and depression caused by reserpine, with “certain” on the WHO-UMC causality category and “probable” on the Naranja scale (8 out of 13 points).

### Pathophysiology

Reserpine is an irreversible inhibitor of the presynaptic vesicular monoamine-transporter (VMAT), and acts by preventing vesicle uptake and hence synaptic release of the monoamine neurotransmitters dopamine, serotonin and norepinephrine. Reserpine binding to VMAT1, found in peripheral neuroendocrine cells, underlies its anti-hypertensive effect, while the sedative and antipsychotic effects of reserpine are mediated by its binding to VMAT2 in the central nervous system. The binding affinity of Reserpine to VMAT2 is three times greater than that to VMAT1 (8).

Ayurveda recommends the use of “sarpagandha” (Rauwolfia serpentina; English: “snakeroot”) for a variety of afflictions, including as a tranquilizer for nervous disorders (9). After being isolated from the root of Rauwolfia serpentina in 1952, reserpine became the first drug to successfully treat hypertension. Mainly due to its sedative, but also to its mild anti-psychotic effects, it was also widely used in the 1950s for psychosis, but then discontinued after Parkinsonism and depression were reported as side-effects (8). The observation that mice dramatically recovered from a reserpine-induced sedative state when given L-Dopa served as the foundation of modern PD therapy (10).

Although PD is unknown to animals in the wild, it is interesting to note that animal models based on low and chronic dosages of reserpine more strongly resemble the phenotype and pathophysiology of PD than genetic models (which account for only 5–10% of PD in humans), or toxin-based models (e.g., MPTP, 6-OHDA) that induces dopaminergic cell death (11). Furthermore, VMAT2-deficient mice have an accumulation of the signature PD protein α-synucleine, and mimic non-motor symptoms of PD, such as a deficit in olfactory discrimination, delayed gastric emptying, altered sleep latency, and depression (12).

[^123^I]FP-CIT is a ligand of DAT, a presynaptic membrane-bound transporter protein responsible for the re-uptake of dopamine from the synaptic cleft. DAT acts in conjunction with intracellular VMAT2 to control the concentration of dopamine at the synapse. Indeed, reserpine has been shown to alter DAT in rodents (13). Furthermore, quantitative positron emission tomography studies indicate that *in vivo* striatal DAT expression is down-regulated in the face of dopamine deficiency, as observed in PD (14). In line with this, DAT availability in our patient markedly increased (by about 25–30%) and ultimately normalized after the reserpine-induced depletion in dopamine was ameliorated. To our knowledge, this is the first report of a reversible decrease in DAT availability due to reserpine toxicity in a human patient.

According to the product information, normalin® contains 15 mg and serpina® 4 mg of root extract per tablet. The concentration of reserpine in roots of Rauwolfia serpentina can be as high as 3% (15), but the actual content of reserpine in commercial preparations is considerably lower, ranging from 0.02 to 0.08 mg (16). Even considering the fact that the patient in the present case took four tablets of serpina® a day during the 6 months preceding his first presentation, his symptoms started 3 years before and progressed continually already when taking a potential maximum of 0.16 mg/day (two tablets/day of normalin® for 1 year initially, then two tablets/day of serpina® over 1.5 years). In the literature, a daily dose of up to 0.25 mg/day is repeatedly mentioned as being safe, but this number is only based on a few historical studies on hypertension that lack the sufficient data to evaluate potential adverse effects (17). The present case suggests that a harmful dose might be much lower than this.

The Ayurvedic physician presumably recognized the rest and postural tremor as side-effects of normalin® and instead prescribed lower-dosed serpina®, along with the herbs ashwaganda (Withania somnifera) and brahmi (Bacopa mannieri), which Ayurvedic medicine recommends for PD (5).

The patient took 3 months to fully recover after discontinuing the Rauwolfia tablets. This is likely attributable to the long half-life of reserpine (up to 168 h) (18), as well as the time needed to replenish VMAT2: a single high dose of reserpine (5 mg/kg s.c.) in rats inhibits the function of striatal VMAT2 for at least 30 days (19). The low-dose dopaminergic and serotonergic medication taken in the initial weeks following reserpine discontinuation was apparently insufficient in overcoming the neurotransmitter deficit.

We did not find similar descriptions of transient parkinsonism after use of reserpine-containing herbs in literature. The main reason that the causality did not reach the highest category on the Naranja scale was that a rechallenge with the herbal drugs themselves or with a placebo was not performed for obvious ethical reasons.

### General Implications

Herbal supplements are not regulated like regular prescriptions. In the USA, for example, they do not require approval and the FDA has to prove that a particular supplement is unsafe before it can be removed from the market. In the European Union, the EMA allows market introduction after a supplement has been used in a traditional setting for at least 30 years, as long as bibliographic data provide sufficient safety data and plausible efficacy.

Due to the lack of regulation in place, the purity of herbal preparations can also be a concern. The proportion of ingredients may vary considerably, depending on factors such as soil quality, fertilization, local climate conditions, time of sowing and harvest, the parts of the plants used (e.g., roots or leaves), and the method of processing (e.g., extraction with water or alcohol) (20). In addition, contamination with pesticides or heavy metals, or even clandestine adulteration, are not uncommon (21).

It also has to be kept in mind that plants potentially contain multiple ingredients that affect the absorption, metabolism, distribution and excretion mechanisms of pharmaceuticals. For example, St. John's Wort, one of the most commonly used herbal medications (2), induces expression of the cytochrome P450 enzyme CYP3A4, responsible for metabolizing up to 75% of drugs, in turn leading to reduced drug effectiveness. On the other hand, patients taking prescription medicines should be told to abstain from grapefruit juice, a strong inhibitor of CYP3A4 (22).

Current trends will likely lead to a further increase in the general use of herbal medicines. The global market, with an estimated worth of US$ 73 billion in 2018, is expected to reach US$ 111 billion by 2023 (23). In 2013, the WHO adopted a 10-year strategy document that was financially supported by China and aimed “to promote the safe and effective use” of T&CM “through the regulation of products, practices and practitioners” and its “integration into national health systems” (24).

However, it could take at least decades to investigate in randomized controlled trials the enormous range of herbal medicines for their efficacy, safety and side-effects (1). In the meantime, especially in western countries, physicians and patients alike should be aware that herbal medicines contain active substances that are sometimes beneficial, but can also have serious side-effects, and hence should be included in the medication history.

## Informed Consent

Written informed consent was obtained from the patient for the publication of this case report.

## Data Availability

No datasets were generated or analyzed for this study.

## Author Contributions

MR: concept, drafting, and revising the manuscript. PM: drafting and revising the manuscript, figure composition.

### Conflict of Interest Statement

The authors declare that the research was conducted in the absence of any commercial or financial relationships that could be construed as a potential conflict of interest.

## Abbreviations

MPTP: 1-Methyl-4-phenyl-1,2,3,6-tetrahydropyridin
6-ODH: 6-Hydroxydopamine.
